# Supplementary material for: Collateral assessment on magnetic resonance imaging/angiography up to 30 hours after stroke onset
Source: PLoS One. 2024 Sep 3;19(9):e0309779. doi: 10.1371/journal.pone.0309779 (PMC11371231; doi:10.1371/journal.pone.0309779)
Supplement: S1 Table — (DOCX) [file pone.0309779.s001.docx]

S1 Table. Multiple regression analysis of core volume at follow-up image

|  | Coefficients | Standard error | t Stat | P value | Lower 95% CI | Upper 95% CI |
| --- | --- | --- | --- | --- | --- | --- |
| Internal carotid artery occlusion | 48.74 | 24.79 | 1.97 | 0.056 | -1.41 | 98.90 |
| PCA laterality positive | 69.01 | 25.47 | 2.71 | 0.01 | 17.49 | 120.53 |
| High hyperintense vessel sign | -86.30 | 24.61 | -3.51 | <0.01 | -136.09 | -36.52 |
| Thrombolysis | -12.76 | 26.42 | -0.48 | 0.63 | -66.20 | 40.67 |

CI; confidence interval

F-test<0.01

Adjusted R-squared=0.40
