# Supplementary material for: Collateral assessment on magnetic resonance imaging/angiography up to 30 hours after stroke onset
Source: PLoS One. 2024 Sep 3;19(9):e0309779. doi: 10.1371/journal.pone.0309779 (PMC11371231; doi:10.1371/journal.pone.0309779)
Supplement: Attachment — Submitted filename: Response to reviewers.docx [file pone.0309779.s002.docx]

**Response to Reviewers**

We thank the reviewers for their insightful comments, which have helped us significantly improve our manuscript. We hope that our revised manuscript is acceptable for publication.

**Reviewer 1**

**Major comments**
*1. It seems strange that there were no patients in the dataset who had undergone mechanical thrombectomy (MT), as there were already 5RCTs on MT in 2015 when this study was conducted. The inclusion criteria seem to include patients who would be good candidates for MT. Please clarify if any of the excluded patients had undergone MT or if there were any study design or institutional limitations.*

**Response:** Thank you for your comment. In explanation, at that time we only had a single neurointerventionist at our centre, and so not all patients were able to be offered treatment. We described the included patients in the ‘population and data collection’ part as follows.

“We included patients who had acute large vessel occlusion (internal carotid artery (ICA) isolated occlusion or tandem occlusion, or MCA segment 1 or segment 2 proximal portion, without effective recanalization between baseline and follow-up imaging.”

To make it clearer, we’ve added the following sentence after the above part.

“Patients with M3 or distal occlusion or those with ICA/MCA occlusion successfully revascularized by thrombolysis or thrombectomy were excluded.”

*2. In this report, it was reported that HVS and PCA laterality were assessed by two readers. Does this mean that two readers evaluated different patients or two readers evaluated the same patients, and if the latter, how were the discrepant cases handled?*

**Response:** Thank you for your comment. Two readers evaluated the same patients. To clarify that part, we’ve changed the following sentence from

“Two readers (S.T.; stroke neurologist, >10 years of experience and Y.T.K.; neurosurgeon, >5 year-experience of stroke clinical practice) graded the HVS and PCA laterality independently with discordance settled by a separate consensus reading.”

to

“Two readers (S.T.; stroke neurologist, >10 years of experience and Y.T.K.; neurosurgeon, >5 year-experience of stroke clinical practice) each graded the HVS and PCA laterality for each patient, independently and regraded by consensus if the grading differed.”

*3. Prestroke mRS should be reported because of the prognostic impact in the HVS high group and the HVS low group. The aetiology of stroke (e.g. TOAST classification) should also be included as the dataset is characterised by a relatively high number of ICA tandem occlusions. Time from onset to imaging should also be included in the table, as it may be related to HVS and PCA laterality.*

**Response:** We agree with you. We’ve calculated the proportion of premorbid mRS 0–1 and compared it between the high HVS and the low HVS group as well as between the PCA laterality positive group and the negative group separately. The results are as follows.

The high HVS group vs. the low HVS group: 19 (86%) vs. 22 (96%), p=0.35

The PCA laterality positive group vs. the PCA laterality negative group: 21 (95%) vs. 23 (88%), p=0.61

We’ve made a new column for premorbid mRS 0–1 and put the above results in the Table 2.

We didn’t evaluate the aetiology of stroke in this study because it’s challenging to accurately determine the aetiology of internal carotid artery occlusion. Instead, we showed the proportion of AF in each group, suggesting the aetiology of cardioembolic stroke.

For the time from onset to imaging, we divided it into time from onset to baseline CT and time from onset to follow-up MRI. We showed them in the Table 2. There was no significant difference in either time between the HVS high or low group and between the PCA laterality positive or negative group.

***Minor comments*** *1. Please describe the management of patients with M3 or more distal occlusions.*

**Response:** Patients with M3 or more distal occlusions were excluded in this study. We’ve added the following sentence in the ‘population and data collection’ part.

“Patients with M3 or distal occlusion or those with ICA/MCA occlusion successfully revascularized by thrombolysis or thrombectomy were excluded.”

*2. The scoring method for HVS seems to be the same as in ref. 11; it is advisable to add this to the METHOD.*

**Response:** We agree with you. We’ve added the following sentence, cited the ref. 11 and changed the following sentence in the method section.

“We adopted the same scoring system in the previous paper [11]. The score was based on a rostrocaudal extension of HVS.”

**Reviewer 2**

**Major comment**

*I recommend conducting an adjusted analysis with the core volume on follow-up scans (and other clinically meaningful aspects) as the dependent variable, incorporating the presence of HVS, PCA laterality, and clot location, especially since the positive PCA laterality group reportedly had a higher incidence of ICA occlusions. This approach would help clarify the independent effects of each variable on patient outcomes, given that the current results are based on unadjusted comparisons.*

**Response:** Thank you for your comment. We’ve performed a multiple regression analysis with the core volume on follow-up as the dependent variable, incorporating the HVS, PCA laterality, clot location and thrombolysis. PCA laterality was related to larger core volume at follow-up, while high HVS was associated with the smaller one. S1 Table is the result of the multiple regression analysis.

S1 Table. Multiple regression analysis of core volume at follow-up image

|  | Coefficients | Standard error | t Stat | P value | Lower 95% CI | Upper 95% CI |
| --- | --- | --- | --- | --- | --- | --- |
| Internal carotid artery occlusion | 48.74 | 24.79 | 1.97 | 0.056 | -1.41 | 98.90 |
| PCA laterality positive | 69.01 | 25.47 | 2.71 | 0.01 | 17.49 | 120.53 |
| High hyperintense vessel sign | -86.30 | 24.61 | -3.51 | <0.01 | -136.09 | -36.52 |
| Thrombolysis | -12.76 | 26.42 | -0.48 | 0.63 | -66.20 | 40.67 |

CI; confidence interval

F-test<0.01

Adjusted R-squared=0.40

In accordance with the above change, we’ve added the following sentence in the statistical analysis section.

“Multiple regression analysis was performed with core volume at follow-up as a dependent variable, incorporating HVS, PCA laterality, occlusion site and thrombolysis as independent ones.”

Then, we’ve added the following sentence in the results section.

“In the multiple regression analysis, PCA laterality positive was related to larger core volume at follow-up, while high HVS was associated with the smaller one. (S1 Table).

***Minor comments***

*1) Page 2, Line 21, “a late time window up to 30 hours”. I assume the “late time window” means between 4.5h and 30h from the onset of the stroke. Please define the starting point of this “late time window” for clear understanding.*

**Response:** We agree with you. We’ve changed that part as follows.

“stroke patients with HVS or PCA laterality between 4.5 hours and 30 hours after stroke onset,”

*2) Page 2, Line 34, “TIMI grade”. I think TIMI was the typo of modified TICI score. However, if this was legitimate, please provide a reason why the scoring system for coronary revascularization was used to evaluate the result of cerebral thrombectomy.*

**Response:** Thank you for your comments. We’ve changed that part as follows.

“Assessment of recanalization was made using the modified thrombolysis in cerebral infarction (mTICI) grade, with assignments of 0 (no recanalization), 1 (minimal recanalization), 2a (partial recanalization with less than 50%), 2b (partial recanalization with more than 50%) and 3 (complete recanalization). Effective recanalization was defined as mTICI 2b to 3.”

*3) For readers who are not familiar with MIStar, please consider adding a brief description of what Delay Time is and how it differs from Mean Transit Time and Time to Peak.*

**Response:** We agree with you. We’ve added the following sentence in that part.

“DT is an index of time to the peak of the residual function, similar to Tmax.”

**Reviewer 3**

*1.When considering the association between HVS and clinical outcome, the possibility of loss of HVS due to the completion of infarct at the same territory should be considered. This study showed the association between high score of HVS and good clinical outcome, but the volume of ischemic core was significantly larger in the low HVS group both at the initial visit and at follow-up. Significant and strong correlation between the low score of HVS and the large ischemic core is not surprising. What is the significance of using HVS rather than initial ischemic core as a predictor of clinical outcome? To elucidate the significance of HVS on clinical outcome, the effect of infarct core volume should be considered.*

**Response:** Thank you for your comments. We’d like to emphasize the usefulness of HVS in clinical practice because small ischemic core may be difficult to recognize on DWI image and the volume may not be immediately calculated. Prominent HVS is easy to recognize on FLAIR image and may assist decision making to progress to EVT. We’ve changed the following part in the discussion section

From

“The clinical implication is that HVS appears to remain useful for interpretation of collateral status, beyond 4.5 hours after stroke onset. Prominent HVS suggesting good collateral supply, in addition to clinical-core mismatch, may assist decision-making to progress to EVT, in hospitals where acute perfusion imaging analysis is not available.”

To

“The clinical implication is that HVS appears to remain useful for interpretation of collateral status, beyond 4.5 hours after stroke onset. Small ischemic core may be difficult to recognize on DWI image and the volume may not be immediately calculated. Prominent HVS suggesting good collateral supply is easy to recognize on FLAIR image, and in addition to clinical-core mismatch, it may assist decision-making to progress to EVT, in hospitals where acute perfusion imaging analysis is not available.”

*2.If the follow-up imaging performed within 30 hours after onset (median 20 hours) was used as surrogate for initial assessment of late presenting stroke, as the author mentioned as the limitation of study, the effect of acute treatment, especially intravenous thrombolysis, could not be ignored. Moreover, in this study, the final core volume at the follow-up would correspond to the initial ischemic core in late presenting stroke. If this study was designed for late presenting stroke, true ischemic core measured after follow-up visit should be analyzed. Similarly, clinical baseline parameters including initial NIHSS, were less meaningful. The author should present the NIHSS score obtained at follow-up.*

**Response:** We agree with you. However, this study was not designed for ‘true’ late presenting stroke but designed for evaluation on MR image using ‘hypothetical’ late presenting stroke. The data was reviewed retrospectively, and information of true ischemic core was not available. We showed the NIHSS score assessed at 24─72 hours after stroke onset in Table 2. Further follow-up NIHSS score was not available because of the retrospectivity one.

Once again, we would like to thank the reviewers for their valuable comments, which have helped improve our manuscript.
